# SPINKs in Tumors: Potential Therapeutic Targets

**DOI:** 10.3389/fonc.2022.833741

**Published:** 2022-02-11

**Authors:** Chengcheng Liao, Qian Wang, Jiaxing An, Minglin Zhang, Jie Chen, Xiaolan Li, Linlin Xiao, Jiajia Wang, Qian Long, Jianguo Liu, Xiaoyan Guan

**Affiliations:** ^1^ Department of Orthodontics II, Affiliated Stomatological Hospital of Zunyi Medical University, Zunyi, China; ^2^ Oral Disease Research Key Laboratory of Guizhou Tertiary Institution, School of Stomatology, Zunyi Medical University, Zunyi, China; ^3^ Microbial Resources and Drug Development Key Laboratory of Guizhou Tertiary Institution, Life Sciences Institute, Zunyi Medical University, Zunyi, China; ^4^ Department of Gastroenterology, Affiliated Hospital of Zunyi Medical University, Zunyi, China; ^5^ Department of Gastroenterology, Affiliated Baiyun Hospital of Guizhou Medical University, Guiyang, China; ^6^ Department of Urology, The Third Affiliated Hospital of Zunyi Medical University, Zunyi, China

**Keywords:** SPINKs, tumor, KLKs, PSTI, uPA, EGFR

## Abstract

The serine protease inhibitor Kazal type (SPINK) family includes SPINK1-14 and is the largest branch in the serine protease inhibitor family. SPINKs play an important role in pancreatic physiology and disease, sperm maturation and capacitation, Nager syndrome, inflammation and the skin barrier. Evidence shows that the unregulated expression of SPINK1, 2, 4, 5, 6, 7, and 13 is closely related to human tumors. Different SPINKs exhibit various regulatory modes in different tumors and can be used as tumor prognostic markers. This article reviews the role of SPINK1, 2, 4, 5, 6, 7, and 13 in different human cancer processes and helps to identify new cancer treatment targets.

## Introduction

Serine protease inhibitors were first found in animal serum and are widely present in plants, animals, bacteria and viruses (1). According to their sequence, number of disulfide bonds and three-dimensional structure, serine protease inhibitors can be classified into at least 18 nonhomologous families. Among them, serine protease inhibitors Kazal type (SPINKs) are one of the more conserved families (2). The family was named after Kazal, who first discovered pancreatic secretory trypsin inhibitor (PSTI) (3). SPINKs have been described in bird eggs, insects, crayfish, and mammalian tissues (such as semen vesicles, pancreas, submandibular glands) and body fluids (such as blood and saliva) and are closely related to hemagglutination, fibrinolysis, embryogenesis, ontogeny, food digestion, inflammation and the immune response (4). At present, more than 100 Kazal-type protease inhibitors have been discovered, but their specific structure and function have not been fully studied, mainly due to the high evolutionary pressure placed on protease inhibitors, which makes the active site highly variable (5). The SPINK family includes members SPINK1-14 and is the largest branch in the serine protease inhibitor family, with each member containing one or several Kazal domains (6–12). The typical Kazal domain is usually composed of 50-60 amino acid residues, including 6 cysteine residues and a relatively conserved sequence. The cysteine residues form three pairs of disulfide bonds (Cys I-Cys V, Cys II-Cys IV, and Cys III-Cys VI) that stabilize the molecular conformation of the domain. The Kazal domain also includes a P1 active site, which is a key site that determines the specificity of protease inhibitors, generally the second amino acid residue after the second cysteine (13).

SPINKs prevent the imbalance of protease activity by regulating serine proteases (14), and are believed to limit enzyme activity in the pancreas and reduce the risk of pancreatitis (15). Due to the formation of a covalent bond between the active lysine carboxyl group of SPINKs and the catalytic serine residue of trypsin, when incubated together, SPINKs and trypsin form a stable but reversible interaction (16). In this way, the activity of trypsin is only temporarily inhibited and may be reactivated in the intestine to promote digestion. Importantly, human SPINKs can inhibit trypsin or chymotrypsin, while SPINKs of other species cannot effectively inhibit human trypsin (17). Kallikrein-related peptidases (KLKs) are the key to epidermal barrier function, participate in the proteolysis of the desquamation process and are valuable tumor expression markers (18, 19). A variety of protease inhibitors, including irreversible serine protease inhibitors (serpins) and SPINKs, have been described to interact with KLKs (18). Therefore, SPINKs are also considered to be closely related to the pathophysiology of the epidermis. In addition, SPINKs are also considered to be related to the structure of epididymal tubules and sperm phenotype (20). Importantly, SPINKs have been reported to occur in human tumors. A relationship between SPINK1, 2, 4, 5, 6, 7, and 13 and tumors has been reported, especially for SPINK1, 5, 6, and 7. SPINK9 may have a potential role in tumors, so it is also discussed.

## The Regulatory Network of SPINK1 in Tumors

The human *SPINK1* gene is located on chromosome 5 and consists of approximately 7.5 kilobases, including 4 exons (21). The SPINK1 protein is 56 amino acids long and contains 6 cysteine residues. These residues form 3 disulfide bridges. This conformation produces a thermally acid-stable tricyclic clover structure (22). SPINK1 is also known as a tumor-associated trypsin inhibitor (TATI) or PSTI (23). Physiologically, SPINK1 is secreted by pancreatic acinar cells as the first line of defense against premature activation of trypsinogen in the acinar and pancreatic duct. SPINK1 is an effective inhibitor of trypsin 1 and 2 but not an effective inhibitor of trypsin 3 (24). In addition to trypsin, SPINK1 also inhibits plasmin, urokinase-type plasminogen activator (uPA) and acrosin, but its inhibitory effect on the first two enzymes is far less than that on trypsin (25). SPINK1 regulates tissue repair, gastric protection, normal pancreas development and other functions (26). SPINK1 regulates tissue repair, gastric protection, normal pancreas development and other functions. SPINK1 is considered to be related to poor overall survival rates of glioma and head and neck, liver, breast, ovarian, lung, pancreatic and renal cancer and good overall survival rates of colorectal and urothelial cancer (27–30). SPINK1 is related to the good progression-free survival of ovarian cancer, but it indicates poor progression-free survival of gastric cancer (26). More importantly, SPINK1 has a profound effect on tumor cell proliferation, metastasis, drug resistance, stemness and differentiation (23, 27).

### The IL-6/STAT3/SPINK1 Signaling Axis Promotes Tumor Progression

**Figure 1:**
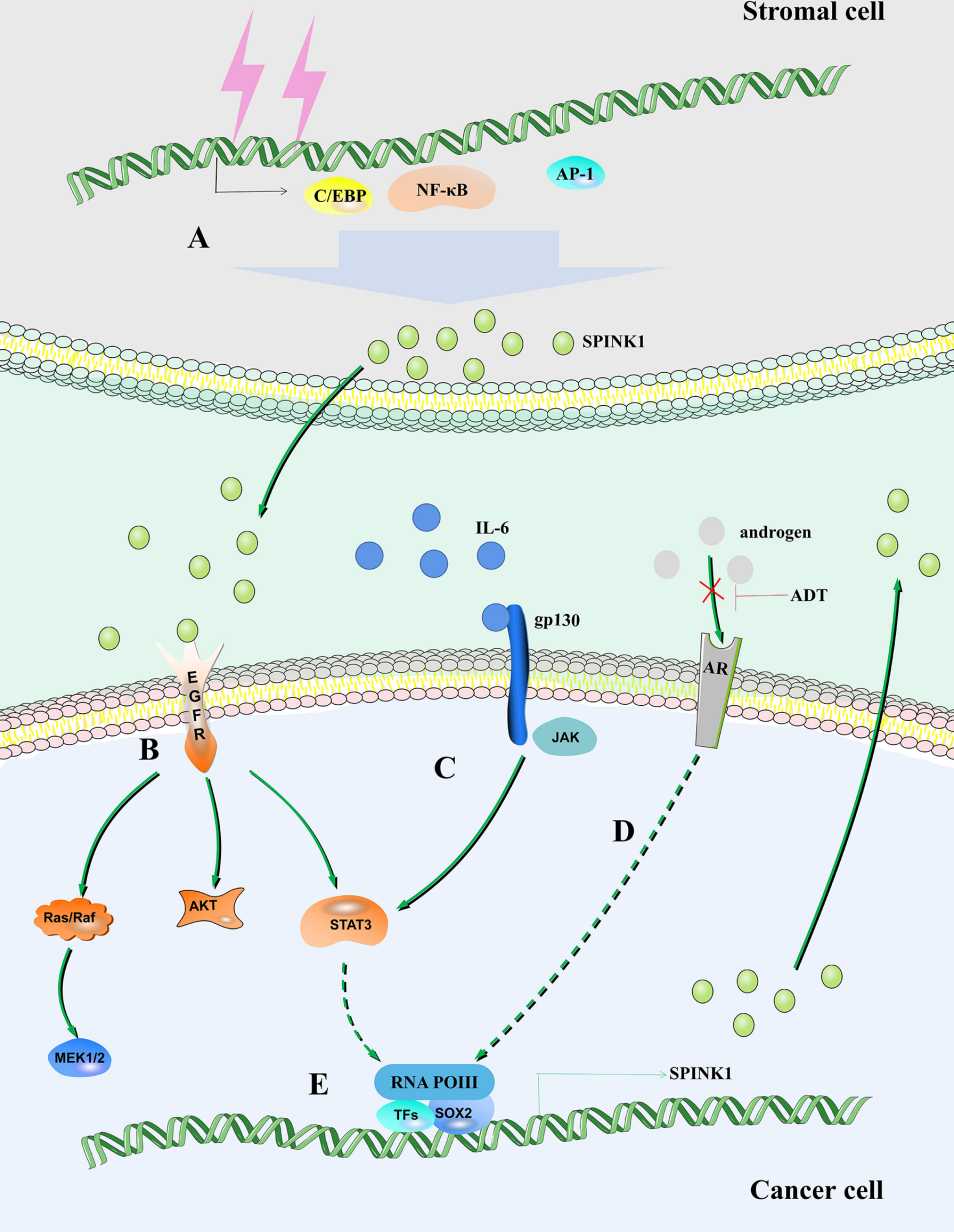
SPINK1 regulates the fate of tumor cells. **(A)** DNA damage in stromal cells leads to increased expression of SPINK1 mediated by NF-κB, activating protein-1 (AP-1) and C/EBP. **(B)** SPINK1 binds to EGFR to activate the EGFR signaling pathway. **(C)** IL-6/STAT3 axis regulates the expression of SPINK1. **(D)** Androgen receptor regulates the expression of SPINK1. **(E)** SOX2 is recruited to the SPINK1 promoter leading to SPINK1 upregulation.

Interleukin 6 (IL-6) is produced by a variety of cell types in the tumor microenvironment (TME), including tumor-infiltrating immune cells, stromal cells and tumor cells themselves (31–33). IL-6 plays an important role in tumor cell growth and survival, angiogenesis, immune regulation of the TME, stromal cell activation, and ultimately disease progression (34). IL-6 directly acts on hepatocellular carcinoma (HCC) cells to induce the expression of signal transducer and activator of transcription 3 (STAT3) target genes, which encode proteins (including IL-6) and drive tumor proliferation and/or survival (35). The expression of the SPINK1 gene depends on the existence of two regulatory regions, including the IL-6 response element and AP-I binding site (36, 37). IL-6 produced in ovarian clear cell carcinoma (OCCC) cells stimulates the common tumorigenic gene expression pattern by regulating the expression of SPINK1 to promote tumor peritoneal metastasis, anoikis resistance and proliferation (38). IL-6 activates SPINK1 by activating the typical STAT3 pathway. Increasing STAT3 phosphorylation promotes the expression levels of SPINK1 and trypsin 1 and 2 (**Figure 1**) (39).

### SPINK1 Is Regulated by miRNA

The expression pattern of microRNAs (miRNAs) can be used as a tool for cancer diagnosis and prognosis, and miRNAs play a role in almost all aspects of tumor biology, including proliferation, apoptosis, invasion, metastasis, and angiogenesis (40). SPINK1 overexpression enhances resistance to gemcitabine (GCB) (a first-line chemotherapy drug for pancreatic cancer) (41). The oleanolic acid (OA) derivative K73-03 reverses SPINK1-mediated GCB resistance by downregulating miR-421 (41). miR-421 is a tumor-promoting factor (42–44). In pancreatic cancer, K73-03 upregulates miR-421 and downregulates SPINK1 through epigenetics, thereby inhibiting mitochondrial function and inducing autophagy and apoptosis (41, 45).

Bhatia et al. (46) found that miR-338-5p and miR-421 are epigenetically silenced in SPINK1-positive prostate cancer. In SPINK1-positive prostate cancer cells, forced ectopic expression of miR-338-5p and miR-421 eliminated tumorigenic properties, including cell cycle progression, stem cell resistance and drug resistance, and the miR-338-5p/-421/SPINK1 pathway may be a valuable target for tumor therapy (46, 47). SPINK1 in prostate cancer is considered to be a potential target of miR-32. miR-32 is an miRNA regulated by the androgen receptor (AR), which is overexpressed in castration-resistant prostate cancer, and miR-32 can promote tumor cell growth and aggressiveness *in vitro* (48, 49).

Circular RNAs (circRNAs) are a new class of noncoding RNAs that play an important regulatory role in physiological and pathological processes. Compared with linear RNA, circRNAs have a special structure that provides them with unique characteristics, such as protection from exonuclease digestion and cleavage and a longer half-life (50). circRNAs retained in the cytoplasm act as miRNA sponges to regulate the expression of miRNA target genes (51). Lin et al. (52) showed that circRPS16 acts as a miR-876-5p sponge to regulate the expression of SPINK1 in HCC. In HCC, miR-876-5p acts as the target of lncRNA PITPNA-AS1, long noncoding RNA SNHG14 and LINC-ROR, thereby regulating HCC proliferation and sorafenib sensitivity (53). The expression of SPINK1 is an independent predictor of the overall survival of HCC and is related to the proliferation, migration and invasion of HCC cells. Gene set enrichment analysis (GSEA) suggests that the metabolism of glycine, serine, threonine and bile acid may be a potential mechanism by which SPINK1 promotes HCC (54). SPINK1 can be used to distinguish well-differentiated HCC from high-grade dysplastic nodules. In one study, the sensitivity of SPINK1 for detecting early HCC was even higher than that of alpha fetoprotein (AFP) (55). In addition, serum SPINK1 has high effectiveness in diagnosing hepatitis B virus (HBV)-related HCC, indicating a poor prognosis (56–58).

### The TME Regulates SPINK1 Expression

The metabolic crosstalk between different tumor compartments leads to an adverse tumor hypoxic environment (TME) and ultimately damages the fitness and effector function of immune cells (59). Several characteristics, including hypoxia, immune status, metabolism, acid status, innervation, and mechanical status, influence the complex and influential nature of the TME (60). Hypoxia causes an increase in secreted SPINK1 levels in a hypoxia-inducible factor-1 (HIF-1)-dependent manner. SPINK1 protein was detected in and around the hypoxic area of tumor tissue, and it increased with decreasing oxygen supply (61). Even under normoxic conditions, secreted SPINK1 protein enhances the radiation tolerance of cancer cells in a manner dependent on epidermal growth factor receptor (EGFR) and nuclear factor erythroid 2 related factor 2 (NRF2) and accelerates the growth of tumors after radiotherapy. SPINK1 secreted by hypoxic cells protects surrounding and relatively oxygenated cancer cells from radiation in a paracrine manner (61).

Clinical radiotherapy and chemotherapy remove tumors by removing rapidly expanding malignant cells, but their off-target effects often cause irreparable damage to benign stromal cells and lead to typical cellular senescence (62). This process is accompanied by the aging-related secretory phenotype (SASP) (63). The SASP eliminates damaged cells by promoting wound healing, tissue repair and immune cell replenishment, thereby promoting tissue homeostasis (64). A variety of soluble factors released by human stromal cells that form the SASP after genotoxic stress, including SPINK1. *In vivo*, SPINK1 is expressed in the stroma of solid tumors and is routinely detected in the peripheral blood of cancer patients after chemotherapy (65). DNA damage mediates the expression of SPINK1 through nuclear factor-κB (NF-κB) and CCAAT/enhancer-binding protein (C/EBP) in the prostate stromal cell line PSC27, and paracrine SPINK1 promotes the invasiveness and chemotherapy resistance of cancer cells. SPINK1 reprograms the expression profile of cancer cells, resulting in a significant epithelial−endothelial transition (EET) (**Figure 1**) (65). A study proposed that prostate cancer can be divided into different molecular subtypes, including mutually exclusive cancers with positive E-26 transformation-specific (ETS) gene fusion, overexpression of SPINK1, and E-cadherin deletion. Patients can be stratified, and different management strategies can be adopted (66).

After androgen deprivation therapy (ADT), aggressive AR-independent neuroendocrine prostate cancer (NEPC) appears (67). SPINK1, which is highly expressed in prostate cancer, is transcriptionally inhibited by AR and its corepressor, REST, while AR antagonists alleviate this inhibition and lead to upregulation of SPINK1. In addition, AR antagonists enable SRY-box transcription factor 2 (SOX2) to bind to the SPINK1 promoter and positively regulate the expression of SPINK1 (**Figure 1**) (68).

### SPINK1 Regulates the EGFR/MAPK Signaling Pathway in Tumors

EGFR is a transmembrane glycoprotein belonging to the ErbB family of RTKs. After binding to ligands, EGFR is activated, triggering subsequent activation of intracellular signaling pathways, such as the mitogen-activated protein kinase/extracellular signal-regulated kinase (MAPK/ERK), phosphatidylinositol 3-kinase/V-akt murine thymoma viral oncogene homolog (PI3K/AKT), and JAK/STAT pathways. These pathways are involved in the proliferation, differentiation, migration and apoptosis of certain cells (69–71). The sequence of human epidermal growth factor (EGF) is similar to that of human SPINK1, and SPINK1 has 50% gene sequence homology with EGF (72, 73). SPINK1 has a spatial structure similar to EGF, and both have a similar number of amino acid residues (56 and 53, respectively). The molecular weight of SPINK1 is approximately 6 kD, and there are 3 intrachain disulfide bridges (74, 75). However, although both SPINK1 and EGF contain 6 cysteines, including 3 disulfide bonds, comparison of their structures reveals completely different protein folds (76). It has been reported that rat monitoring peptide (the rat homolog of human SPINK1) competes with mouse EGF to bind to EGFR in Swiss 3T3 fibroblasts (74). Chang et al. (77) observed that SPINK1 interacts with EGFR and promotes hepatocyte proliferation. An EGFR-blocking antibody eliminates the migration-promoting effect of SPINK1 on human colon cancer HT-29 cells (78). SPINK1 protein binds to EGFR and activates its downstream signals, leading to the progression of pancreatic cancer, prostate cancer, bladder cancer (BCa), pancreatic ductal adenocarcinoma and breast cancer cells (**Figure 1**) (79–83). Although the binding affinity of SPINK1/EGFR is lower than that of EGF/EGFR, EGFR and its downstream molecules, STAT3, AKT, and ERK1/2 are phosphorylated by SPINK1 (79, 80).

Mammalian MAPKs include c-Jun NH2 terminal kinase (JNK), p38 MAPK and ERK. These enzymes are serine-threonine protein kinases that regulate various cell activities, including proliferation, differentiation, apoptosis, survival, inflammation, and innate immunity (84). SPINK1 can promote the proliferation of rat normal hepatocytes through the p38, ERK and JNK pathways (77). SPINK1 promotes a tumorigenic phenotype of colorectal cancer (CRC) by activating the PI3K/AKT and MAPK/ERK signaling pathways, and SPINK1-positive WiDr cells are sensitive to AKT and MEK inhibitors (85). In addition, SPINK1 promotes the motility and epithelial-mesenchymal transition (EMT) of HCC cells through the MAPK and ERK pathways, resulting in increased vimentin expression and decreased E-cadherin expression (86). EGFR abnormally overactivates the downstream cancer-promoting MAPK signaling pathway. In view of the direct activation effect of EGFR by SPINK1, the activation of MAPK by SPINK1 may be at least partially mediated by EGFR.

### SPINK1 and PTEN Expression Are Mutually Exclusive in Prostate Cancer 

The fusion of E-26 transformation-specific-related gene (ERG) and transmembrane serine protease 2 (TMPRSS2) genes is the most common genomic change in prostate cancer. ERG is an oncogene that encodes members of the ETS transcription factor family. TMPRSS2 is an androgen-regulating gene that is preferentially expressed in the prostate. Most of the less frequent ETS fusion partners are also androgen-regulated and prostate-specific (87–89). SPINK1 expression and ETS fusion status are mutually exclusive, and SPINK1 outlier expression is an independent predictor of biochemical recurrence after prostate cancer resection. SPINK1 plays an important role in ETS rearrangement-negative prostate cancer (90–92). SPINK1 and ERG are expressed in 25% and 42.7% of primary prostate cancer cases, respectively. A total of 91.7% of patients with primary prostate cancer were observed to have varying degrees of loss of phosphatase and tensin homolog (PTEN), of whom 54.2% exhibited complete loss. In primary prostate cancer, the SPINK1^+^/ERG^-^ phenotype accounts for 12.5%, the SPINK1^+^/ERG^+^ phenotype accounts for 16.7%, the SPINK1^-^/ERG^+^ phenotype accounts for 25.0%, and the SPINK1^-^/ERG^-^ phenotype accounts for 45.8% of cases, and all prostate cancers expressing SPINK1 or ERG show PTEN deletion (93). Bismar et al. (94) further confirmed that PTEN deletion is significantly related to ERG rearrangement, AR amplification and SPINK1 overexpression. None of the SPINK1-overexpressing tumors showed AR amplification or PTEN expression (95). PTEN is a phosphatase that metabolizes PIP3 (the lipid product of PI3K), which directly counteracts the activation of the tumorigenic PI3K/AKT/mTOR signaling network. Loss of PTEN tumor suppressor function is observed in many types of cancer and is one of the most common events (96). However, whether SPINK1 inhibits PTEN expression still needs more direct evidence.

## SPINK2 Indicates Poor Tumor Prognosis and Inhibits the Progression of Testicular Cancer

The SPINK2 protein contains a typical Kazal domain consisting of six cysteine residues, forming three disulfide bridges. The P2-P2'(Pro(23)-Arg(24)-His(25)-Phe(26)) active site of the SPINK2 protein may be the key to its molecular functions (97). SPINK2 is synthesized in the testis, epididymis and seminal vesicles but not in the prostate (95). The expression of SPINK2/3 is critical for the quality and physiological function of sperm (98–101). In addition, one of the most significant effects of SPINK2 deficiency is rupture of the Golgi apparatus, which is a key organelle for protein processing and transport, especially of membrane proteins (8). The expression of SPINK2 is closely related to the development of cancer, and high levels of SPINK2 transcripts can be detected in patients with primary skin follicular center cell lymphoma (102). Upregulation of SPINK2 gene expression in patients with acute myeloid leukemia is associated with poor prognosis (103, 104). SPINK2 is significantly elevated in most of the leukemia cell lines studied and plays an important role in tumor progression and response to treatment (97). The European Organization for Cancer Research and Treatment (EORTC) divides primary skin large B cell lymphoma (PCLBCL) into two categories: primary skin follicular center cell lymphoma (PCFCCL) and PCLBCL of the legs (PCLBCL-leg). Due to differences in prognosis and preferred treatments, it is important to distinguish between PCFCCL and PCLBCL-leg. Hoefnagel et al. found that SPINK2 is highly expressed in PCFCCL compared to PCLBCL-leg, so it may be used as a marker for differential diagnosis.

**Figure 2:**
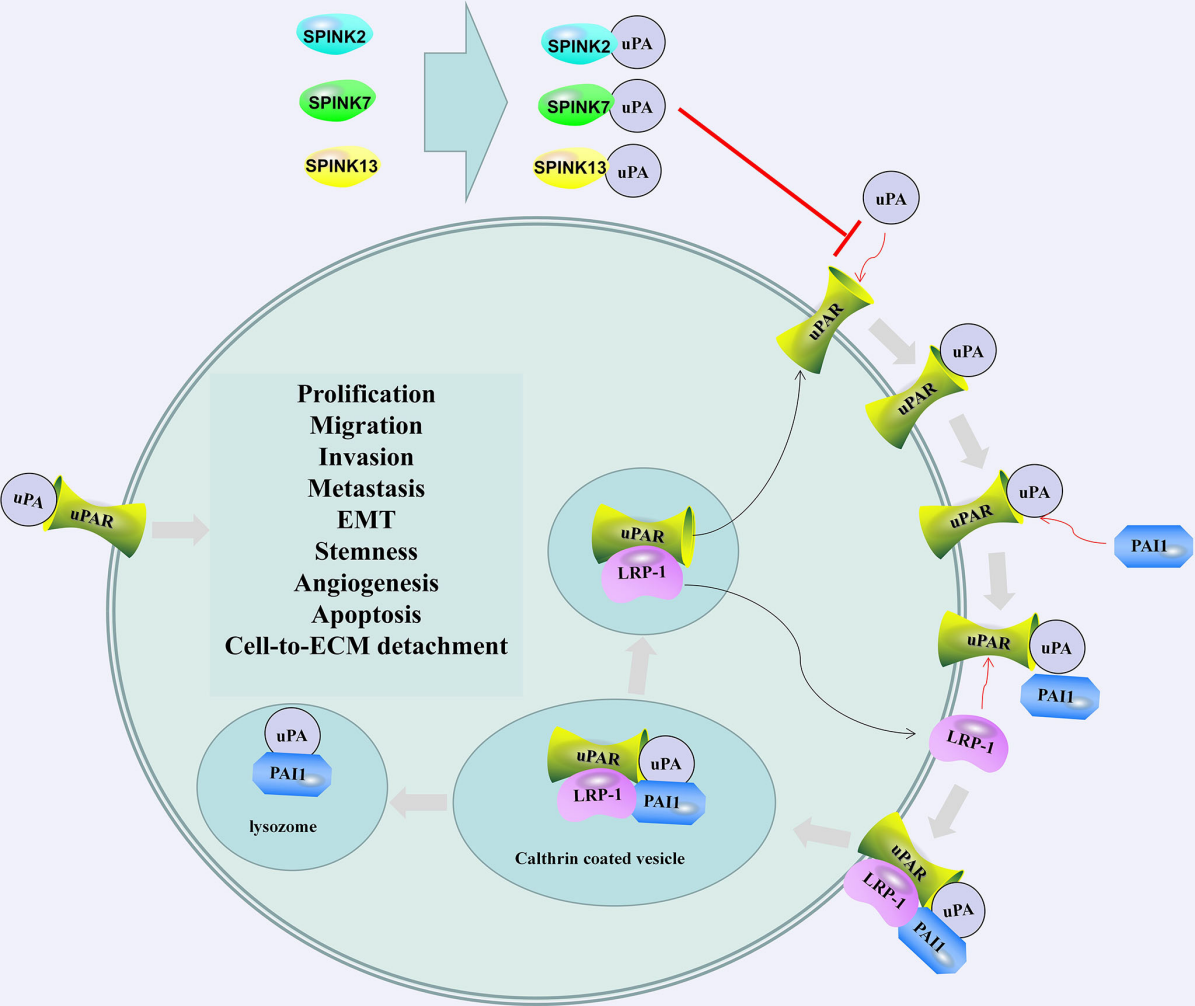
SPINK2/7/13 and plasminogen activator inhibitor 1 (PAI1) regulates the activation and regulation of uPA/uPAR. SPINK2/7/13 interacts with uPA and hinders the formation of uPA/uPAR complex. The combination of PAI1 and uPA/uPAR led to the internalization of the entire assembly. uPA and PAI-1 are degraded in the lysosome, and Low-density lipoprotein (LDL)-related protein-1 (LRP-1) and uPAR are recycled into the membrane. The recovered uPAR can be combined with the available uPA (106). After activation, uPA can cause the degradation of the extracellular matrix and promote cell prolification, migration, invasion, metastasis, EMT, stemness, and angiogenesis.

In the testes, the presence of SPINK2 not only affects sperm quality but also is associated with testicular cancer. Tazarotene-induced gene 1 (TIG1), also known as retinoic acid receptor responder 1, is one of the genes highly upregulated by tazarotene in skin raft culture (105). TIG1 and SPINK2 are downregulated in testicular cancer tissues. TIG1 inhibits the invasion, migration and EMT of NT2/D1 cells. SPINK2 enhances the regulation of uPA activity and EMT inhibition by TIG1. The interaction between TIG1 and SPINK2 plays an important role in inhibiting the EMT of testicular cancer cells by downregulating the uPA/uPA receptor (uPAR) signaling pathway (**Figure 2**) (107). The combination of uPA and uPAR helps to activate plasminogen and convert it into plasmin, which in turn triggers a series of proteolytic cascades to degrade extracellular matrix components, thereby causing tumor cell EMT (**Figure 2**) (108).

## SPINK4 Can Serve as a Prognostic Marker for CRC, BCa and Barrett's Esophagus

SPINK4, also known as PEC60, was originally isolated from the intestines of pigs (109) and is mainly expressed in the gastrointestinal tract and immune system (110). The SPINK4 gene of human chromosome 9p13.3 encodes a precursor protein consisting of 86 amino acids. The precursor protein consists of 26 amino acids, which are characterized by a C-terminal cysteine, an N-terminal glutamate and a cell secretion mark. A total of 60 residues of, are believed to be involved in the defense against degradation of mucosal and epithelial tissue proteins (111). In the colon and Barrett's esophagus, SPINK4 markers appear before morphologically identifiable goblet cells, which may help identify the early stages of intestinal metaplasia (111). Previous studies have found that the expression of serum SPINK4 in patients with CRC is elevated, and this increased expression has high diagnostic value (112). Research by Chen et al. (113) further showed that the high expression of SPINK4 is related to the advanced clinicopathological characteristics and poor treatment response of rectal cancer patients receiving chemotherapy. In contrast, Wang et al. (114) believed that the downregulation of SPINK4 at the mRNA and protein levels is related to the poor prognosis of CRC patients and a high TNM stage. In multivariate regression, SPINK4 was confirmed as an independent indicator of low survival in CRC patients (115). In addition, SPINK4 is related to the prognosis of BCa, reflecting the good overall survival rate of BCa (116).

SAM tip domain ETS factor (SPDEF) can regulate the terminal differentiation and maturation of intestinal goblet cells (117). Treatment of LS174T colon cancer cells with a Notch/γ-secretase inhibitor blocked SPDEF and inhibited the expression of anterior gradient 2 (AGR2), mucin 2 (MUC2), resistin-like beta (RETNLB) and SPINK4 (118). Notch signaling can regulate the expression of SPINK4 in middle-ear epithelial cells (115). It is suggested that Notch is the upstream regulatory factor of SPINK4. The current research on SPINK4 and CRC is mainly based on bioinformatics analysis or has drawn conclusions based only on a small number of human samples. In addition, the results of different scholars are contradictory. Therefore, more research is needed to confirm the diagnostic and targeted therapy value of SPINK4 in CRC.

## SPINK5 Can Serve as a Tumor Prognostic Marker and Inhibits Tumor Progression Through Multiple Mechanisms

The *SPINK5* gene is located in the 5q32 region and consists of 15 functional regions, encoding a 15-domain classical and nonclassical Kazal inhibitor called lymphoepithelial Kazal-related inhibitor (LEKTI) (119). KLKs are expressed in the epidermis from the stratum spinosum layer to the stratum corneum layer, while SPINK5 is expressed on the surface of the stratum granulosum near the stratum corneum (120). LEKTI D6-D9 regulates the activity of trypsin, subtilisin A, KLK5 and KLK7 (121–124). All the tested fragments of LEKTI (D5, D6, D8, D9, D11 and D15) had no inhibitory effect on human KLK3, KLK8, cathepsin G, trypsin, elastase, plasmin and thrombin. D8 and D11 had the strongest inhibitory effects on KLK5 and KLK14, while their inhibitory effects on KLK7 were weaker. D5, D6, D9, and D15 were weaker inhibitors of KLK5, KLK7 and KLK14 (125). In another study, recombinant LEKTI (rLEKTI) D1 and D6, rLEKTI D6 and D9, and rLEKTI D9 and D12 were shown to inhibit KLK5 and KLK14 and, to a lesser extent, KLK6 and KLK13 (126). Loss of SPINK5 function leads to increased epidermal protease activity, leading to premature division of desmosomes (in the stratum corneum), abnormal maturation of filaggrin and protease activator receptor-2 (PAR-2) activation (127–129). SPINK5 deficiency leads to Netherton syndrome (NS) characterized by impaired skin barrier function, epidermal hyperplasia, hair abnormalities, chronic skin inflammation, and atopic dermatitis (130–133). Cases of NS syndrome complicated by tumors have been reported many times (134–136). In fact, the lack of SPINK5 is indeed closely related to tumor progression, especially that of squamous cell carcinoma.

Compared with that in normal esophageal tissue, the expression level of SPINK5 mRNA and protein in esophageal cancer tissue (including squamous cell carcinoma tissue) is significantly lower and predicts tumor lymph node metastasis and differentiation (137–139). Similar to the case in esophageal cancer, SPINK5 is downregulated in 85.7% of head and neck squamous cell cancer (HNSCC) cases and is an independent prognostic predictor for HNSCC patients (140–143). An increased KLK5/SPINK5 mRNA ratio is associated with shorter HNSCC overall survival (144). Moreover, coexpression of KLK5 and KLK7 is associated with a low survival rate of non-human papilloma virus (HPV)-associated oral squamous cell carcinoma (145). In addition, the expression of SPINK5 in the urine of patients with transitional cell carcinoma of the bladder was higher than that in the control group. The upregulation of SPINK5 expression combined with the expression of nuclear receptor subfamily 0 group B member 1 (NR0B1), retinoic acid early transcription-1 (RAET1E), and secreted phosphoprotein 1 (SPP1) is associated with liver cancer vascular invasion, histological grade, clinical stage, and T stage, suggesting that the poor prognosis of liver cancer is associated with an immunosuppressive tumor microenvironment (146). However, the role of SPINK5 as a specific protein for the diagnosis of BCa needs to be further studied (147).

Glycogen synthase kinase-3 (GSK-3) is an evolutionarily conserved serine/threonine kinase that is ubiquitously expressed in mammalian eukaryotic cells. In addition to regulating the activity of glycogen synthase (GS), GSK-3β can also act on many signal protein structural proteins and transcription factors to regulate cell differentiation, proliferation, survival and apoptosis (148). SPINK5 can inhibit GSK3β phosphorylation and promote β-catenin protein degradation, thereby inhibiting the proliferation, migration and invasion of esophageal cancer cells (142). Li et al. (149) found that SPINK5 can promote the apoptosis of gastric cancer cells by downregulating the expression of B cell lymphoma-2 (BCL-2) and upregulating the expression of BCL-2-associated X (BAX) and NF-κB. G9a is a histone lysine 9 (H3K9) methyltransferase, involved in the formation of heterochromatin, DNA methylation and transcriptional silencing (150, 151). SPINK5 is one of the downstream target genes of G9a. G9a downregulates the expression of SPINK5 through the methylation of H3K9me2, thereby promoting the proliferation, migration and invasion of renal cell carcinoma (RCC) cells (152). Research by Liu et al. (48) showed that miR-32 is an AR-regulated miRNA that is overexpressed in castration-resistant prostate cancer, and miR-32 may promote the growth of prostate cancer cells *in vitro* by downregulating the expression of SPINK5.

The Hippo pathway is a key regulator of tissue growth and consists of the MST1/2 and LATS1/2 kinase cascades, as well as the downstream effectors and transcriptional coactivators YAP and TAZ. The controlled transcription program involves cell proliferation, survival, migration, stemness, and differentiation (153). The YAP1/TAZ-TEAD transcription network is essential for maintaining skin homeostasis and affects the proliferation and differentiation of squamous cells (154). In HNSCC, SPINK5 inhibits the expression of KLK5/PAR-2/IL-8 and the activation of the YAP1-TAZ/TEAD transcription network, thereby inhibiting matriptase-dependent carcinogenic effects (155). The inhibitory effect of SPINK5 on KLK5 and the activation of the Hippo pathway may be important factors controlling the molecular landscape of the occurrence and development of squamous cell carcinoma.

## SPINK6 Can Serve as a Tumor Prognostic Marker and Interacts With the EGFR Protein to Promote Tumor Progression

SPINK6 is expressed in the stratum corneum and the inner root sheath of hair follicles (156–158). Unlike LEKTI, SPINK6 has only one typical Kazal domain (159). SPINK6 crosslinks with the transglutaminases of human keratinocytes and the human epidermis and maintains inhibitory activity to protect specific substrates of KLKs (160). Recombinant SPINK6 has inhibitory effects on KLK4, KLK5, KLK6, KLK12, KLK13 and KLK14 in the nanomolar to subnanomolar range, but it has an inhibitory effect on KLK1, KLK3, KLK8, KLK11, thrombin, plasmin, matriptase, prostaglandin, columnar inhibition of cell lyase, cathepsin G, neutrophil elastase and chymotrypsin (159, 161). Tumor necrosis factor-α (TNFα)/IFNγ and all-trans retinoic acid expression and mechanically induced skin barrier dysfunction all lead to the downregulation of SPINK6 expression (159).

It has been reported that SPINK6 is cross-linked with fibronectin *via* transglutaminase, and it has been shown to protect fibronectin from the cleavage of KLK5 (159). Since fibronectin is a recognized EMT marker, SPINK6 may also play an important role in EMT regulation (162). As a secreted protein, SPINK6 promotes the metastasis of nasopharyngeal carcinoma cells through autocrine and paracrine mechanisms. In addition, SPINK6 induces EMT by binding to EGFR and activating EGFR and downstream AKT signals (163). Importantly, the elevated expression of SPINK6 in primary nasopharyngeal carcinoma is an independent adverse prognostic factor for overall survival, disease-free survival and distant metastasis-free survival of patients (164). SPINK6 is upregulated in HNSCC and is thought to be able to predict the risk of death from HNSCC (165). SPINK6 is involved in the biochemical recurrence of patients with prostate cancer 3 years after prostatectomy (166). SPINK6 has been observed to be expressed in colorectal malignancies, but the significance of SPINK6 expression is still unclear (167).

## The Diagnostic and Therapeutic Value of SPINK7

SPINK7, also known as esophageal cancer-related gene 2 (ECRG2), is expressed in the fetal skin, thymus, esophagus, oral epithelium, thyroid, brain, lung, heart, stomach, liver, spleen, colon, kidney, testis, muscle, gallbladder and adult esophageal mucosa, while SPINK7 is significantly downregulated in primary esophageal cancer tissue (167). Current research shows that SPINK7 plays an important role in skin homeostasis, inflammatory skin diseases, inflammatory bowel disease, eosinophilic esophagitis and esophageal inflammatory responses (164, 168–170).

### Diagnostic Value of SPINK7

Yue et al. (171) found that SPINK7 short tandem repeats (STRs) are a genetic susceptibility factor for esophageal squamous cell carcinoma (ESCC), and SPINK7 TCA3/TCA3 alleles may play a role in the development of ESCC. In the northern Indian population, the SPINK7 TCA3/TCA4 genotype is associated with the risk of esophageal cancer (172). Analysis of STRs in SPINK7 exon 4 in 86 patients with complete surgical resection found that the TCA3/TCA3 and TCA3/TCA4 genotypes both accounted for 47% of cases, the TCA4/TCA4 genotype accounted for 7% of cases, and the TCA3/TCA3 genotype was significantly associated with reduced survival rates (173). The STR TCA3/TCA3 polymorphism in exon 4 of SPINK7 is associated with poor recurrence-free survival in patients with OSCC with complete surgical resection and may be a potential prognostic indicator (174). The incidences of the ECRG2 STR TCA3/TCA3 genotype in pancreatic cancer and pancreatitis patients were 51% and 50%, respectively, those of TCA3/TCA4 were 40% and 54 (46%), and those of TCA4/TCA4 were 9% and 4%. However, the SPINK7 STR polymorphism is not significantly correlated with a reduced risk of pancreatic cancer or overall survival (175). SPINK7 is highly expressed in chromophobe RCC, but the SPINK7 mRNA level is downregulated in the saliva of patients with gastric cancer (176, 177).

### SPINK7 Inhibits Tumor Progression Through the uPA/uPAR Pathway

Promoting apoptosis and direct binding with uPA may be the mechanism by which SPINK7 inhibits tumors (164, 178). SPINK7 and uPA form a complex that changes the dynamic relationship between uPAR and beta1 integrin and destroys and inhibits the Src/MAPK pathway, thereby inhibiting cell migration and invasion. In contrast, the loss of SPINK7 significantly enhanced the association between uPAR and beta1 integrin, increased the basal activation of the Src/MAPK pathway, and stimulated the migration and invasion of human fibrosarcoma cells and breast cancer cells (179). SPINK7 can bind to uPA with molecular weights of 55 and 33 kDa, reducing the proteolysis of the plasmin substrate d-v-ph-lys-p-nitroaniline and inhibiting the growth of cancer cells by downregulating uPA/plasmin activity (**Figure 2**) (180). Using isotope-labeled SPINK7 to perform heteronuclear magnetic resonance experiments, it was found that the uPA binding loop of SPINK7 was consistent with the reaction site loop of the serine protease in the Turkish egg mucus third domain (OMTKY3), which may be the binding site of SPINK7 and uPA (181). In addition to uPA, SPINK7 may also interact with metallothionein 2A, metallothionein 1H, metallothionein 1G, ferritin, erythrocyte membrane protein band 4.2, mitochondrial ribosomal protein S12, and hypothetical protein FLJ10101 and regulate cell proliferation, apoptosis and other physiological processes (182).

### SPINK7 Inhibits Tumor Progression Through the p53 Pathway

p53 is a tumor suppressor protein that tightly regulates cell growth by promoting cell apoptosis and DNA repair. After p53 is mutated, it loses its function, leading to abnormal cell proliferation and tumor progression (183). During interphase and mitosis, SPINK7 localizes to centrosomes and centromeres, and participates in centrosome amplification in a p53-dependent manner. The deletion of SPINK7 not only destabilizes p53, downregulates p21, and increases cyclin E/CDK2 activity, thereby initiating centrosome amplification but also eliminates the ability of p53 to localize to the centrosome (184). SPINK7 plays an important role in ensuring centrosome replication, spindle assembly checkpoints and accurate chromosome segregation. SPINK7 deletion may lead to chromosome instability and aneuploidy in human cancer (185). Moreover, SPINK7 combined with cisplatin (DDP) therapy can reduce DDP resistance in esophageal cancer and induce apoptosis by upregulating p53 expression and downregulating PCNA and Bcl-2 expression (184, 186). The p53 gene is mutated in approximately 50% of human cancer cells. The p53 mutant protein escapes ubiquitination-dependent degradation, acquires carcinogenic function, and promotes tumorigenesis, malignant progression, metastasis, and chemotherapy resistance (187). In OSCC, the expression of SPINK7 is downregulated, while the expression of p53, RB, NF-κB and CYP4B1 is upregulated. In aggressive OSCC, SPINK7 and human epidermal growth factor receptor 2 (HER2) proteins are lower, while the TP53 and RB1 proteins are increased in less aggressive OSCC. SPINK7, HER2, p53, and RB1 expression changes can be used for molecular staging of OSCC lesions (188).

### The Upstream Factors and Therapeutic Value of SPINK7

The long noncoding RNA (lncRNA)-mRNA regulatory network shows that lncRNA HCG22 is associated with the coexpression of SPINK7 and ADAMTS12 and that SPINK7 is regulated by lncRNA HCG22 in ESCC (189). In addition, miR-1322 can regulate SPINK7 in an allele-specific manner, and the level of miR-1322 in serum can be used as a potential diagnostic biomarker for ESCC patients (190). RNA-targeted therapy provides a platform for drug research and development, and targeting RNA to regulate SPINK7 may be an effective method (191). Song et al. (192) used an adenoviral vector (Ad-SPINK7) as a biological treatment tool and found that Ad-SPINK7 can inhibit the invasion and adhesion of HCC cells at a low titer and change the expression of NF-κB, MMP2 and E-cadherin to reverse the malignant tumor phenotype. During the experiment, no obvious toxic reaction was seen in the model animals. SPINK7 is a potential molecular target in tumor biotherapy strategies.

## SPINK9 as a Potential Factor Impacting Tumors

SPINK9 is strongly expressed in the epidermis of human palms but is not expressed or is expressed at very low levels in non-palmoplantar skin; in addition, it is moderately expressed in the thymus, pancreas, liver and brain and has low or undetectable expression in other tissues. SPINK9 has a strong inhibitory effect on KLK5, and high concentrations of SPINK9 have a slight inhibitory effect on KLK8. SPINK9 may not have an inhibitory effect on other KLKs (193–195). Compared with LEKTI, SPINK9 is a better inhibitor of KLK5 under acidic conditions (196). SPINK9 is an epidermal antimicrobial peptide that can selectively kill Escherichia coli (197). In addition, SPINK9 activates purinergic receptors and promotes metalloproteinase/EGFR-dependent keratinocyte migration (198). Therefore, SPINK9 is considered to be a component of the skin barrier. In fact, some scholars have observed that SPINK9 is expressed not only in healthy palm and plantar skin but also in chronic simple lichens, actinic keratosis and squamous cell carcinoma (199). However, the significance of SPINK9 expression in squamous cell carcinoma is unknown. It is worth noting that KLK5 is associated with vaginal cancer (200), cutaneous squamous cell carcinoma (cSCC) (196), ovarian cancer (201), endometrial cancer (202), ESCC (203), breast cancer (204), prostate cancer (205), uterine cervical cancer (206), colorectal adenoma-carcinoma (207) and ovarian cancer (201). In view of the inhibitory effect of SPINK9 on KLK5, we believe that SPINK9 is a potential tumor-influencing factor.

## SPINK13 Can Serve as a Tumor Prognostic Marker and Inhibits Tumor Progression Through the uPA Pathway

The SPINK13 protein consists of an N-terminal signal peptide and a Kazal domain and is essential in the process of sperm maturation (9). Androgen and its receptor interact with androgen response elements in androgen response genes and regulate the expression of SPINK13 (9). Schrödter et al. (208) SPINK13, SLC6A3, TNFAIP6, NPTX2, NDUFA4L2, ENPP3 and FABP6 were found to be upregulated in RCC (209). Subsequent studies showed that the transcription and protein expression of SPINK13 were significantly increased in clear cell renal cell carcinoma (CCRCC). The increase in SPINK13 mRNA expression was significantly correlated with a decrease in progression-free survival and overall survival. GSEA showed that SPINK13 is involved in the complement, apical junction, EMT, glycolysis, hypoxia and inflammation signaling pathways (210).

uPA promotes the invasion, growth and metastasis of cancer cells by activating matrix metalloproteinases (MMPs), leading to the destruction of the extracellular matrix (211). In HCC cells, SPINK13 interacts with uPA and directly inhibits the cleavage of MMP9 by uPA (**Figure 2**) (212). In ovarian cancer patients, overexpression of SPINK13 is associated with a higher overall survival rate. SPINK13 inhibits cell proliferation, migration and EMT by inhibiting uPA (211).

## Conclusion and Perspectives

SPINKs are members of an ancient gene family and exist in many animals, such as insects, birds and mammals (3, 213). The characteristic features of the SPINK family include at least one conserved Kazal domain and 6 identical cysteines forming a 1-5/2-2/4/3-6 disulfide bond pattern (7). All SPINK members are responsible for regulating the activity of serine proteases and preventing uncontrolled proteolysis (14). However, the biology of SPINKs in normal homeostasis and disease pathogenesis is not fully understood. The relationship between SPINK1, 2, 4, 5, 6, 7, and 13 and tumors has been described, and these proteins have been shown to affect tumor cell proliferation, metastasis, drug resistance, stemness, EMT and other aspects through a wide range of signaling cascades (such as EGFR/MAPK, uPA/uPAR, NF-κB, KLKs, Hippo, and GSK-3β, P53) and have a profound impact.

Considering that SPINK5, SPINK6 and SPINK9 are all clustered on a chromosomal complex of 5q33, they are selective KLK inhibitors. The effect of SPINK5/6 on tumors is largely achieved *via* inhibition of KLK5. We also discussed the possibility that SPINK9 can affect tumors by inhibiting KLK5. In addition, SPINK3 can also inhibit KLKs (214) and activate PI3K/AKT by enhancing the expression of AKT1 to promote the proliferation of normal liver cells (215). Therefore, SPINK3 and SPINK9 have potential biological significance in tumors. However, research on the relationship between SPINKs and tumors is still in its infancy. Further research on the protein structure, expression mode and mechanism of SPINKs will be the basis for targeting these proteins in tumor treatment.

## Author Contributions

CL, XG, QL, and JL had the idea for the article. Literature search was performed by CL and XG. The manuscript was written by CL and JL. JA, QL, and JW critically revised the work. MZ, JC, and XL design and draw the figures in this review. All authors read and approved the final manuscript.

## Funding

This study was supported by the National Key R&D Program (2016YFC1102800); Guizhou Province Medical Biomaterials R&D Talent Base [QianRenLingFa (2018) No. 3]; the Sixth Talent Foundation in Guizhou province (rcjd2019-9); the Graduate Research Fund of Guizhou Province [Qian-Jiao-He YJSCXJH (2019)087]; Zunyi Medical Biomaterials R&D and Innovative Talent Base [ZunWei (2019) No. 69]; the Youth Science and Technology Talents Growth Project of Guizhou Education Department [Qian-Jiao-He KY ZI (2018)236]; Outstanding Young Talent Project of Zunyi Medical University (17zy-002) (F-801); The Project to Cultivate Young Scientific; Master Fund of Zunyi Medical University (S-81); Key Project of Zunyi Science and Technology Bureau Zunyi Medical College Affiliated Stomatological Hospital Combine Fund [ZUN SHI KE HE SHE ZI (2018) 239] and Technological Talents in Colleges and Universities of Guizhou Province [Qian Jiao He KY (2021) 215].

## Conflict of Interest

The authors declare that the research was conducted in the absence of any commercial or financial relationships that could be construed as a potential conflict of interest.

## Publisher’s Note

All claims expressed in this article are solely those of the authors and do not necessarily represent those of their affiliated organizations, or those of the publisher, the editors and the reviewers. Any product that may be evaluated in this article, or claim that may be made by its manufacturer, is not guaranteed or endorsed by the publisher.
